# Patient and Staff Perceptions of Intradialytic Exercise before and after Implementation: A Qualitative Study

**DOI:** 10.1371/journal.pone.0128995

**Published:** 2015-06-12

**Authors:** Hannah M. L. Young, Nicky Hudson, Amy L. Clarke, Maurice Dungey, John Feehally, James O. Burton, Alice C. Smith

**Affiliations:** 1 Leicester Kidney Exercise Team, Academic Unit, Leicester General Hospital, Leicester, United Kingdom; 2 School of Applied Social Sciences, De Montfort University, Leicester, United Kingdom; 3 Department of Infection, Immunity & Inflammation, University of Leicester, Leicester, United Kingdom; 4 School of Sport, Exercise and Health Sciences, Loughborough University, Loughborough, United Kingdom; 5 John Walls Renal Unit, Leicester General Hospital University Hospitals of Leicester NHS Trusts, Leicester, United Kingdom; Hospital Universitario de La Princesa, SPAIN

## Abstract

**Introduction:**

Despite guidance and evidence for the beneficial effects of intradialytic exercise (IDE), such programmes are rarely adopted within practice and little is known about how they may best be sustained. The Theoretical Domains Framework (TDF) was used to guide the understanding of the barriers and facilitators to initial and ongoing IDE participation and to understand how these are influential at each stage.

**Materials and Methods:**

Focus groups explored patient (n=24) and staff (n=9) perceptions of IDE prior to the introduction of a programme and, six months later, face to face semi-structured interviews captured exercising patients (n=11) and staffs’ (n=8) actual experiences. Data were collected at private and NHS haemodialysis units within the UK. All data were audio-recorded, translated where necessary, transcribed verbatim and subject to framework analysis.

**Results:**

IDE initiation can be facilitated by addressing the pre-existing beliefs about IDE through the influence of peers (for patients) and training (for staff). Participation was sustained through the observation of positive outcomes and through social influences such as teamwork and collaboration. Despite this, environment and resource limitations remained the greatest barrier perceived by both groups.

**Conclusions:**

Novel methods of staff training and patient education should enhance engagement. Programmes that clearly highlight the benefits of IDE should be more successful in the longer term. The barrier of staff workload needs to be addressed through specific guidance that includes recommendations on staffing levels, roles, training and skill mix.

## Introduction

There is an increasing focus within healthcare on reducing the burden of chronic disease via lifestyle interventions including exercise. Available guidance recommends exercise counselling be built into chronic disease management strategies given its undisputed efficacy in the management of a diverse range of long-term conditions.[1–3]

Haemodialysis (HD) patients exhibit significantly poorer physical and psychological functioning than matched healthy individuals and other chronic disease populations, leading to greater mortality, higher healthcare utilisation and poorer quality of life.[4–12] Exercise counselling and interventions are particularly warranted in this group of patients. Intradialytic exercise (IDE), using a static bicycle during haemodialysis treatment, offers a solution to many of the health and well-being issues experienced by HD patients and achieves better adherence than other programmes.[5–11,13,14]. However, specific IDE guidelines are ill-defined and rarely adopted in practice [4,15–18]

Lack of implementation of IDE into clinical practice may be influenced by the perceptions of patients and HD staff. Numerous barriers to initiating exercise behaviour have been reported. [19–29] Studies examining these barriers and facilitators have been primarily survey based. [20,25–28] There have been few qualitative studies exploring patient and staff perceptions of exercise and even less investigating IDE specifically. [19,21–23] Available evidence focuses on the initiation of exercise, whilst the factors influencing ongoing participation have received less attention.[24–29]

Psychological theory has been shown to be helpful in understanding why evidence is not implemented and practice and behaviour change interventions driven by theory may be more effective. [30–33] Despite this, only two qualitative studies have used a theoretical approach, the Promoting Action on Research Implementation in Health Services (PARIHS) framework, to guide their understanding of the barriers and facilitators to IDE implementation. [19,22] This framework has been inductively developed, is largely untested. [34] It specifically relates to the initial implementation of an intervention and may not adequately address how practice and behaviour change may be sustained in the longer term. [34].

The Theoretical Domains Framework (TDF) offers a validated, accessible and more comprehensive alternative, which can be used to identify appropriate behaviour change techniques to address barriers to implementation and support the maintenance of a behaviour. [30–33] To date the TDF has been used in many different healthcare settings, including those designed to address lifestyle change (including physical activity levels) in chronic disease. [30–33, 35] The TDF simplifies and integrates multiple psychological and organisational theories relevant to practice and behaviour change in order to make such theory more accessible and usable in practice. The TDF consists of 12 domains identified by consensus approach. Within each domain are several related theoretical constructs relevant to behaviour change. The 12 domains are outlined within Table 1. [30–33]

**Table 1:** Domains with the Theoretical Domains Framework [30].

Domains	
Knowledge	Skills
Social/ professional role and identity	Beliefs about capabilities
Beliefs about consequences	Motivation and goals
Memory, attention and decision processes	Environmental context and resources
Social influences	Emotion
Behavioural regulation	Nature of the behaviour

An outline of the domains included within the Theoretical Domains Framework.

This study was designed to capture patient and staff perceptions before (phase one) and then six months after an IDE programme was established (phase two). The aim was to explore barriers and facilitators to initial and ongoing participation and to understand how these are influential at each stage. The TDF has been used within this study to inform the organisation of data and emphasize ways in which the findings can be used to implement and sustain IDE programmes in practice.

## Materials and Methods

### Study design

The study was approved by the National Research Ethics Service, East Midlands—Northampton (NRES reference number 11/EM/0127) and by The University Hospitals of Leicester NHS Trust Research and Development Department (Ref: 71552). At all stages participants gave written informed consent.

We used a qualitative methodology with a pragmatic approach, which was action-orientated and intended to generate knowledge useful to resolving problems—in this instance providing information that can be used to implement and sustain an IDE intervention.[36]

### Participant recruitment

Participants were recruited from an NHS hospital-based outpatient haemodialysis unit that has 30 dialysis stations and treats 170 patients, and a Fresenius run satellite outpatient unit with 19 stations, treating 114 patients. Both units offer 4 hour shifts over 6 days and are located in a multi-cultural urban setting that serves patients of mixed socio-economic status.

**Fig 1:**
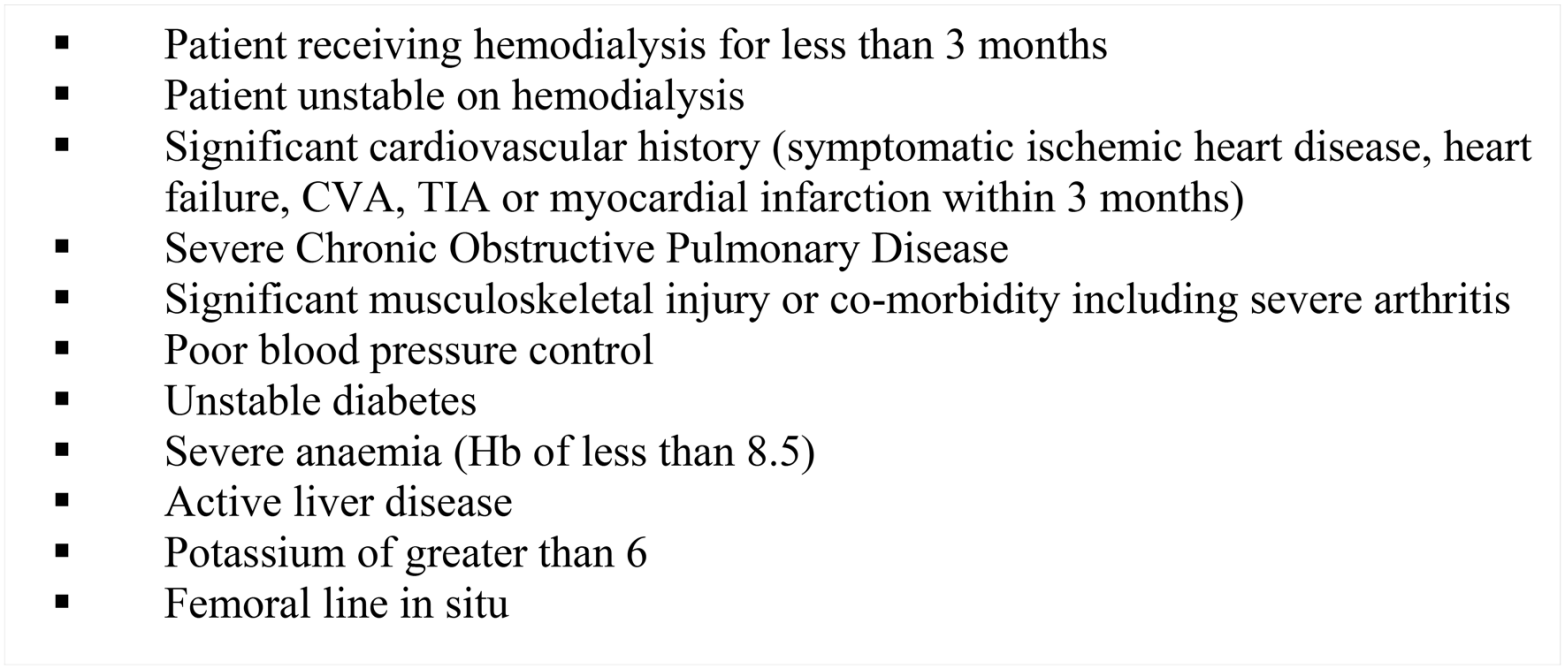
Exercise eligibility criteria. The inclusion criteria applied to the patient participants. It was deemed unreasonable to gather views regarding an exercise programme from patients who were not fit to participate in such a programme.

At phase one as many patients as time allowed over nine shifts (six from the NHS unit and three from the Fresenius unit) selected at random and who did not meet the exclusion criteria (Fig 1) were approached in person regarding the study by their Nephrologist. All staff with regular routine patient contact were eligible for inclusion and were approached in person by the nurse in charge. For phase two all patients and staff with six months of involvement in the exercise programme were approached regarding an interview.

Following an initial expression of interest, purposive, non-probability sampling was used to select participants that could provide a diverse range of perspectives. Following established principles, sampling continued until data saturation was achieved.[36]

### Data collection

Phase one was conducted prior to the implementation of an IDE programme. Focus groups promoted discussion and explored how patients and staff perceived IDE.[29, 30] Focus groups were facilitated by researchers uninvolved in routine patient care (HMLY, NH, ACS). HMLY is a renal research physiotherapist, NH an experienced qualitative researcher with no experience of renal patients, exercise or dialysis. ACS is an senior health researcher and registered exercise instructor with extensive renal experience.

For two patients, whose first language was not English, face to face semi-structured interviews were conducted, as small numbers precluded a focus group. These participants were interviewed in their first language by a professional bilingual translator with health research experience. The translator was provided with specific contextual knowledge prior to the interviews. Patient and staff focus groups were conducted separately, with staff groups further divided by grade to enable free expression and data comparison.[36–38]

**Fig 2:**
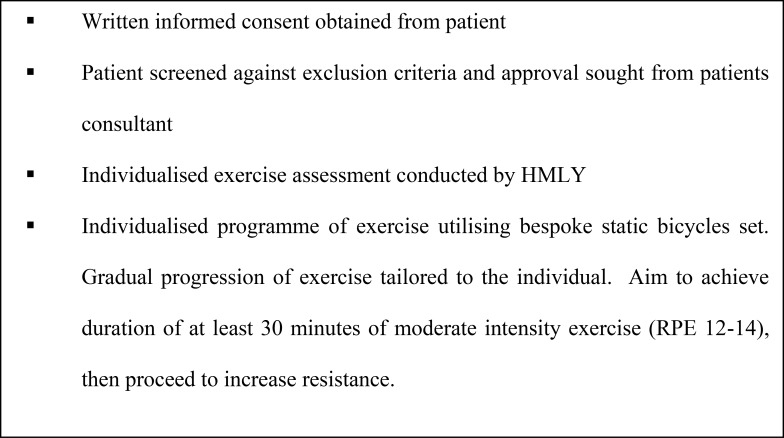
Outline of the IDE programme. An overview of the IDE programme implemented with the satellite unit following phase one of the study.

Following phase one, IDE was introduced at the satellite unit. The programme was designed and implemented by HMLY and MD; an exercise physiologist with renal experience. The programme was offered to all patients who did not meet any of the exclusion criteria (Fig 1). An outline of the programme is provided in Fig 2. Six months later, at phase two, NH and ACS conducted face to face semi-structured interviews with exercising patients and staff, as they had not had not been involved in the delivery of the programme.

**Fig 3:**
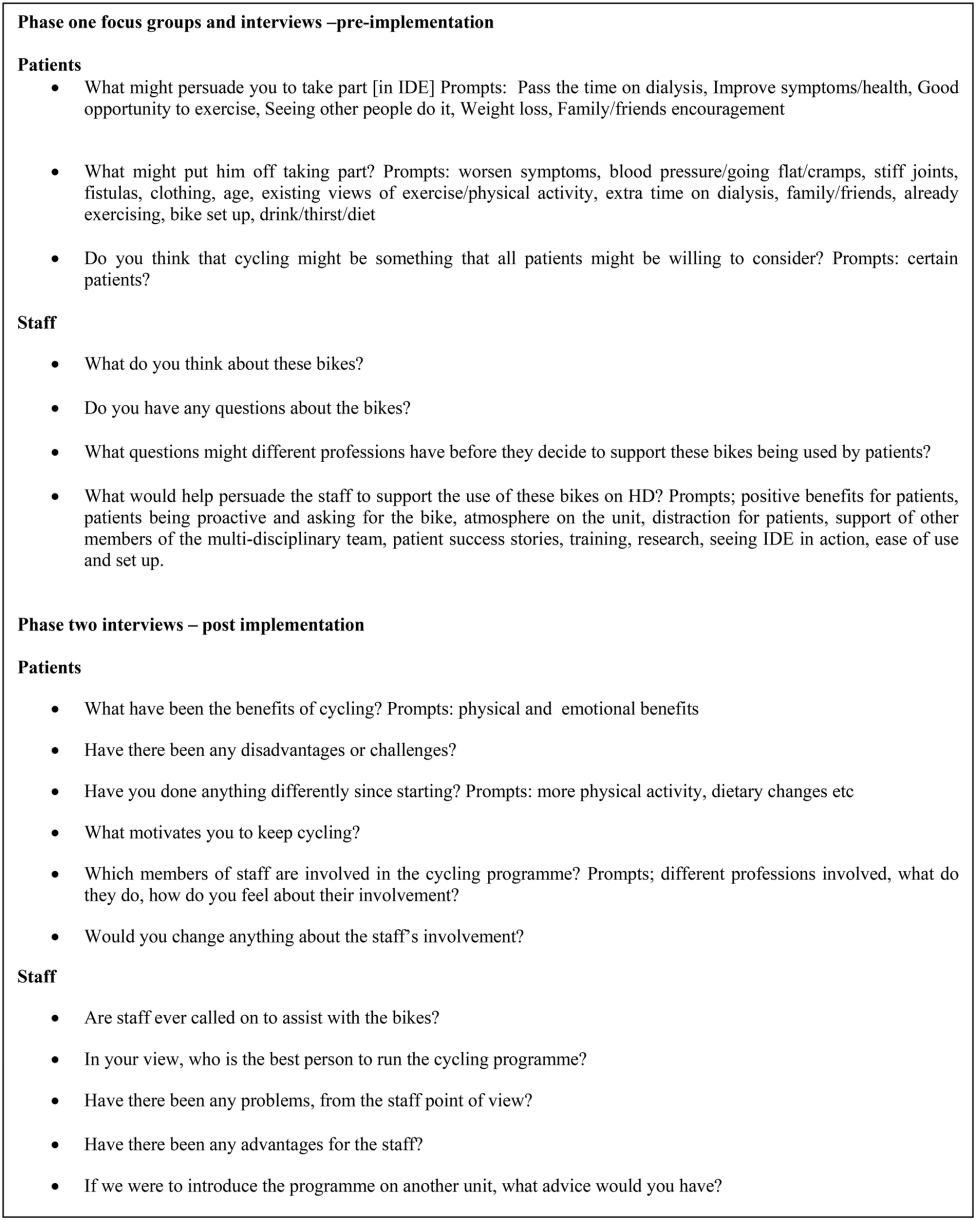
Example questions from focus groups and interviews. Excerpts from the topic guides used for staff and patients in both phases of the study.

Topic guides for focus groups and semi-structured interviews were developed using existing literature and the assistance of a patient and public involvement group, who suggested question prompts and gave views on the data collection methods. Patients from minority ethnic groups were included to enhance cultural competence. Early focus groups and interviews acted as pilots. Fig 3 provides examples of some of the questions and prompts used.

Focus groups and interviews averaged 56 minutes duration and were conducted face to face in neutral settings away from the dialysis units. Post implementation interviews were primarily conducted at patients’ homes and on occasion participants’ spouses were present. Credibility was ensured by summarising the discussion following data collection and asking participants to confirm or add anything.[38]

### Data analysis

All data were audio-recorded, professionally transcribed verbatim and translated prior to analysis where necessary. Field notes were also subject to analysis.

Two researchers (HMLY, ALC) analysed the data using a framework approach. [39–40] ALC is a research psychologist and registered exercise instructor with renal experience. Following familiarization with existing literature on IDE barriers and facilitators and the TDF, a descriptive coding framework was developed that was then systematically applied to the data by HMLY (focus groups) and ALC (interviews). NVivo software (NVivo v9, QSR International, Doncaster, Australia) was used to facilitate data coding. This initial framework focused on barriers and facilitators to IDE initiation and maintenance in staff and patient groups separately. Any emergent codes that did not occur a priori were not forced to fit the coding framework but were added dependent on whether they were a barrier or facilitator.

Both researchers then discussed the findings and created thematic charts which grouped and refined related barriers and facilitators and identified relationships between the two participant groups and the phases of the study [39–40]. These charts were reviewed by the research team and validity was ensured through continuous discussion. Consensus meetings were utilised to discuss and explain the themes as well as any divergent accounts that arose [39–41].

## Results

**Fig 4:**
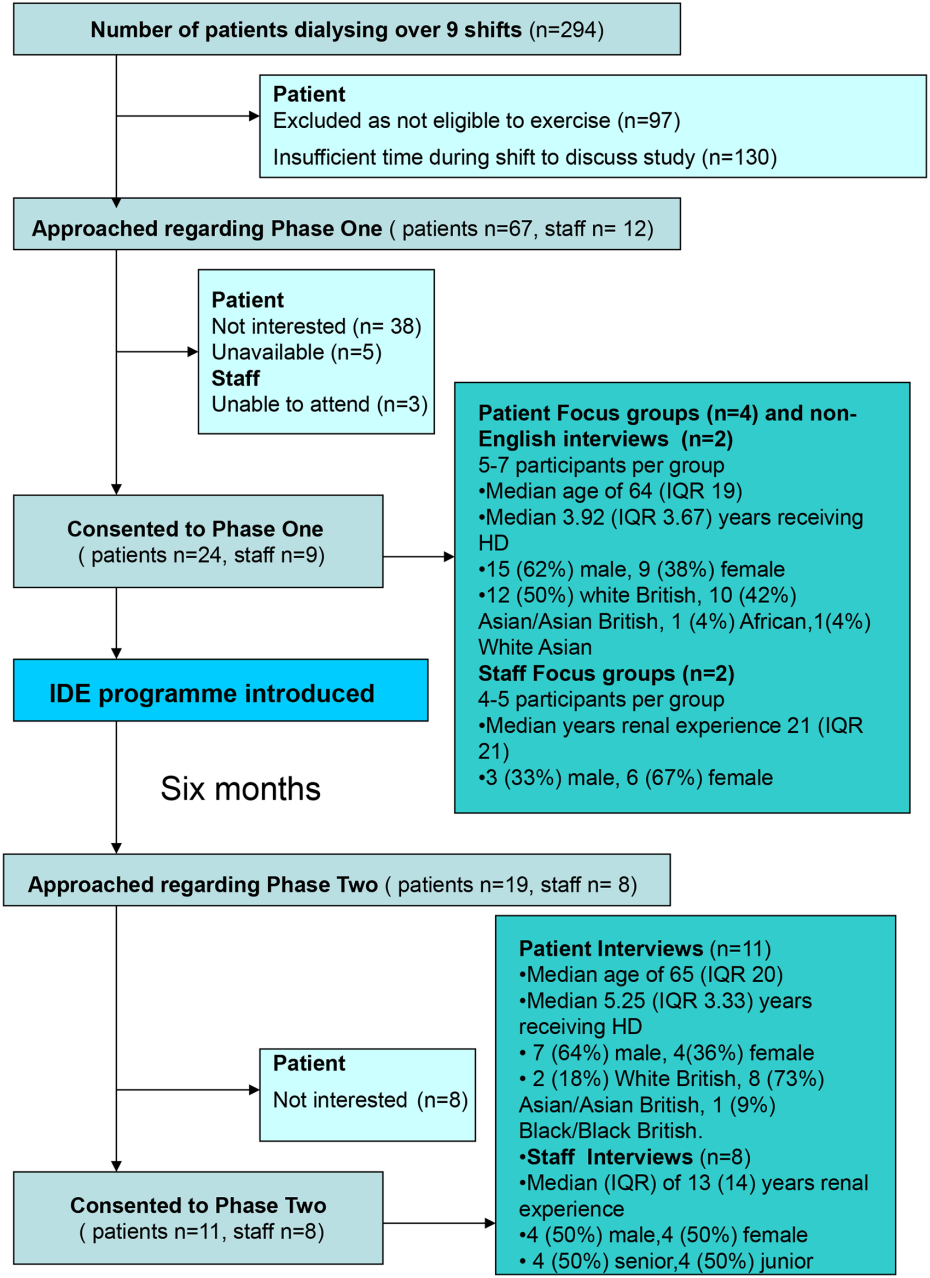
Consort diagram. Flow diagram illustrating participant characteristics and numbers of patients recruited at each phase of the study.

Fig 4 demonstrates the flow of participants through the study and their demographic information. At Phase One, one staff focus group comprised senior nurses, doctors and dieticians (5, 56%), whilst the other consisted of junior nurses and healthcare assistants (4, 44%). At phase two, only 4 patients had previously participated in a focus group.

### Exercise barriers and facilitators

Prior to implementation barriers focused upon environment and resources and also beliefs about IDE which created a range of negative emotions. Facilitators identified included enhancing knowledge and skills in relation to IDE and factors that enhanced participants’ belief about their capabilities. Post implementation, barriers relating to environment and resources remained, together with difficulties over professional roles in relation to IDE. Facilitators to sustaining IDE were related to positive beliefs about participating in IDE and the importance social and professional influences for staff, particularly teamwork and collaboration.

Exemplar quotations are included in Tables 2–3 (pre-implementation) and Tables 4–5 (post-implementation). Our findings are mapped to the TDF within these tables. Themes are presented in order of the frequency by which they were discussed across both patient and staff groups, with frequently discussed themes being described first to give an idea of their importance and influence.

**Table 2:** Pre—implementation barriers, as identified by patients and staff.

Pre-implementation barriers	Theory domains	Patient focus groups	Staff focus groups
**Staff workload**	Beliefs about consequences, environmental context and resources	“If [IDE] requires less intervention on [the] part [of the staff] then it’s a good thing but if they’re being called up more frequently then it’s probably a bad thing.”	“It might not work if we have a very busy period. We have a lot of patients who need a lot of care, that would become our priority and it wouldn’t be the bike.” (Junior staff focus group)
		“My concern is the nurses need to be nursing not lifting bikes on and off beds”	“There will always be an initial staff step back because its more work for them to do” (Senior staff focus group)
		“The whole thing works better if you have [the nurses] co-operation and rather than increasing their work its in the quieter periods so it works for everybody”	
**Patients fears and anxieties**	Beliefs about consequences,optimism, emotions	You’re a little bit more aware of what damage it could do. You’re a little bit scared of the consequences should anything happen.”	
		“I wonder whether it might affect, any movement, and pull the needles out.”	
		“I am more concerned if I have low blood pressure, and pass out I would be stuck on a chair with a cumbersome bike at the end”	
		“If you are on this bike and you fall ill, there is no doctor [at the satellite unit]. You have got the same chance as Jo Public ringing an ambulance.”	
**Staff beliefs about IDE and patients**	Beliefs about capabilities	“We’re all aware of the need to exercise but its time and inclination, if it’s provided for you when you’re trapped [having dialysis] you can’t avoid it.”	“I have only ever heard negatives [about IDE]. I was working in [another region] and they did it there and I heard a lot of moans…and then you see all the bikes at the side not being used” (Junior staff focus group)
		“When it was first mentioned one of the nurses came to me and said they won’t let you do that. I said yes they will, why wouldn’t they? They won’t. And he … didn’t seem too keen”.	“[Patients] don’t really exercise, some do, but the majority don’t.” (Senior staff focus group)
			“At the moment I would say [exercise is] very low on [patients] agenda.” (Junior staff focus group)

Verbatim quotations from participants for each facilitator are identified and ranked in order of the frequency by which they are mentioned across both patient and staff groups. Blank areas indicate that this facilitator was not discussed by that group.

**Table 3:** Pre—implementation IDE facilitators, as identified by patients and staff.

Pre-implementation facilitators	Theory Domains	Patient focus groups	Staff focus groups
**Enhanced knowledge and skills**	Knowledge, Skills, Beliefs about consequences	“I suffer a lot from cramp, would the cycling make any difference to that?”	“A lot of [patients] will come up with… barriers, so if you can have the knowledge about it to be able to overcome those just in a general chat” (Senior staff focus group)
**Assessment**		“One of the problems when you come of dialysis is that… your legs have stiffened up…if it will help with that, brilliant”	“Will we get any training on how to work [the bike]?” (Junior staff focus group)
		“If the BP could improve it might motivate people to take part”	“[Training] gives the staff chance to learn about [IDE] and understand the information and be able to learn it. You can’t expect them to do it straight away.” (Senior staff focus group)
		“I quite enjoy [exercise]…and I tend to push things a bit…but I don’t know now whether that’s a good or a bad thing. I’d like to ask someone”	
		“How long would you exercise for if you did do it?”	
		“I think until you’ve tried it you don’t know, you have to try it”	
**The influences of peers and colleagues**	Social/professional role and identity, beliefs about capabilities, reinforcement, social influences	“People talk and everybody on the units tend to know each other and then say well I had a go on [the bicycle], it’s great, you know, and then more people will come forward I think”	“We should all get shown how to use the machine. That would throw a spanner in the works if people say they didn’t know how to work it.” (Junior staff focus group)
		“I think if patients see the next person cycling they’ll say I cycling they’ll say I could do it”	“I think it’s there persona around the person giving the advice. We all have the knowledge but I think [the patients] might prefer initially to have [an exercise professional].” (Senior staff focus group)
		“If people are negative about it then you’ll get other people oh I don’t want to do it either.”	
**Assessment**	Belief about capabilities, Optimism, Reinforcement, Goals, Memory attention and decision processes, Behavioural Regulation, Emotion	“I think I’d want an ok from my consultant to say that you are fit enough to do it for a start. Because obviously underlying problems again, you know, could make a difference to what you do and how long you do it.”	
		“You want to tailor the thing to your specific needs. You can’t have one size fits all.”	
		“If you know you’re going to get another assessment, you want to be better. It gives you more encouragement to do it and you don’t want to fail. You know it’s going to be monitored so you tend to be a little bit more committed.”	
**Exercise professional support**	Social/ professional role and identity	“I think Physiotherapist would be trained. I do not feel nurse would know much.”	“[An exercise professional] would be specifically coming to do [IDE] and we have got a lot of other things and we can be taken away at any point.” (Junior staff focus group)
		“I would have thought [an exercise professional] ought to set [the bike] up for us really. Set it up properly and tell us what we‘re capable of.”	“I think long term it doesn’t need to be [an exercise professional] that runs it, you might find a champion comes from the most unlikely source really…” (Senior staff focus group)
		“Who’s going to be there to give in-depth advice and answer questions, [an exercise professional] but also fairly clued up as to our problems as renal patients… who can give an honest kind of an answer how far I can go with the exercise bike.”	

Verbatim quotations from participants for each facilitator are identified and ranked in order of the frequency by which they are mentioned across both patient and staff groups. Blank areas indicate that this facilitator was not discussed by that group.

**Table 4:** Barriers to IDE post-implementation, as identified by patients and staff.

Post implementation barriers	Theory domains	Patient	Staff
**Staff workload**	Environmental context and resources	“Well in this situation here and now, we are short of nurses…if you put an extra load on them about exercising, I don’t think that’s possible.” (67 year old male, Asian patient)	“Unless the staffing numbers start to get better I don’t think we are going to have the chance [to assist with IDE], it depends on how busy the shifts are.” (Nurse)
		“Its getting the nurses to [help] and making sure they don’t forget. Either they have forgotten or they are probably shorted staffed.” (57 year old male, Asian patient)	“I think the vast majority of staff are open to the idea of cycling, it’s just what would happen on the particular day. So if the [exercise professional] said on X day we are going to have some training, is the shift going to be a full compliment, are the staff actually going to be in? I don’t think anyone has got a resistance to actually learning.” (Healthcare Assistant)
**Lack of staff responsibility**	Professional role and identity	“For a start you will have to have [an exercise professional] there but once the staff are trained then its fine. That will reassure [patients] and then you can get some dedicated staff to do it.” (65 year old male Asian patient)	It would be nice to have [an exercise professional] across two units or [a dedicated staff member] so it doesn’t just burn out.” (Senior nurse)
			“If you have somebody named on a shift that will [provide the exercise] they can set it up and monitor [the patients].” (Healthcare Assistant)

Verbatim quotations from participants for each barrier are identified and are ranked in order of the frequency by which they are mentioned across both patient and staff groups.

**Table 5:** Facilitators to IDE post-implementation, as identified by patients and staff.

Post implementation facilitators	Theory domains	Patient	Staff
**Positive outcomes of participating in IDE**	Beliefs about consequences, Reinforcement, Emotion	“I used to struggle with my blood pressure, towards the end it always used to drop, I wondered if the exercise would help to stabilise it and it did, so that was a plus” (56 year old male Asian patient)	Last year [before the cycling] the dialysis patients tended to be unmotivated, depressed. I’ve seen them cycling and they are more cheerful, happy, its helping them” (Nurse)
		“I can now walk up to the village which is about half a mile and I feel it’s the cycling that’s helped” (75 year old female White British patient)	“As a doctor working on a dialysis unit it can sometimes be fairly bleak in that dialysis is a very good treatment for keeping people alive but doesn’t always enable people to live. I think if there is a treatment that makes [patients] feel better that makes you feel a whole lot better about what you do to people.” (Consultant Nephrologist).
		“Because of the exercise I can sleep better. I can sleep 5–6 hours at a time” (67 year old male Asian patient)	“I am surprised at a …lady I thought wouldn’t do it but she did. I needed to be proven wrong because we are not always right” (Senior nurse)
**Collaboration and teamwork**	Social influences, Behavioural regulation, Social/professional role and identity		“Even [nurse in charge] will get the bikes out …you have to have her on board, and the two deputies.” (Dialysis Assistant)
			“Because the [exercise professional] has taught [the patients] we just set the bike up for them. Patients will tell us how to fill the paperwork in…they will say come back in however many minutes or if I have a problem I will call you…” (Nurse)

Verbatim quotations from participants for each facilitator are identified and ranked in order of the frequency by which they are mentioned across both patient and staff groups. Blank areas indicate that this facilitator was not discussed by that group.

### Pre-implementation

#### Barriers. Staff workload

Staff and patients both expressed concern about a lack of staff resources and busy workloads within the HD environment. All patients were wary of creating extra work for staff and believed that a lack of time would reduce supervision and encouragement during exercise. Staff agreed, and junior staff in particular strongly believed that IDE would increase in their workload.

#### Patients’ fears and anxieties

Patients were initially unsure of the consequences of participating in IDE which was described as “*going into the unknown*” (Pre-implementation focus group). Patients described a range of emotions about participation including being afraid of disrupting their treatment, particularly through dislodging their needles and injuring themselves. They were fearful that a cumbersome bike may impact on safety should an emergency occur. These concerns were particularly evident in patients dialysing at satellite units and those who had experienced hypotension during treatment.

#### Staff beliefs about IDE and patients

Staff were initially extremely negative regarding IDE, viewing it as a burden to both themselves and patients. They perceived patients to be uninterested in IDE, incapable or unsuitable for exercise particularly if they were older or from a minority ethnic background. This directly contrasted with patient views, who despite their initial anxieties, viewed IDE as an opportunity to overcome exercise barriers and a positive use of treatment time. Patients were aware of staff members’ negative perceptions and felt this could dissuade them from participating.

#### Facilitators. Enhanced knowledge and skill

All patients anticipated a wide variety of potential improvements, primarily reduction in symptoms, better cardiovascular health and confidence. Patients wanted more information about the benefits of IDE and what participation would involve. Patients proposed that an opportunity to ‘try’ IDE without commitment would enhance their confidence, enable informed decision-making and reduce fears. Similarly staff expressed a need for greater knowledge and also requested comprehensive training to enhance their skills around running an IDE programme, particularly setting up the bikes and encouraging patients to participate.

#### The influences of peers and colleagues

Patients described being strongly influenced by their peers and felt that seeing others they viewed as similar exercising on haemodialysis would alleviate their fears and positively influence how capable they felt to participate in IDE. The desire for peer support was particularly relevant for female patients from minority ethnic backgrounds. Patients from two focus groups, however, suggested that any negative experiences may dissuade others. Social influences were also relevant to HD staff. Junior staff strongly stressed the need for training to be available to all grades of staff and professional groups, whilst some senior staff believed they already possessed the knowledge and skills required.

#### Assessment

Although not influential for staff, all patients in the pre-implementation phase felt an exercise assessment, including Nephrologist approval, was imperative prior to embarking on an IDE programme. Patients deemed this assessment necessary to determine safety and for exercise to be tailored in light of their individual co-morbidities, ages and frailties, which were considered to be important determinants of exercise ability. Such assessment was anticipated to further increase self-belief in relation to IDE participation. Ongoing assessment of progress was also anticipated to help maintain long-term motivation by reinforcing the benefits of exercising.

#### Support from a trained exercise professional

Patients felt that assessment should be conducted by a professional with both renal and exercise expertise. Junior staff felt strongly that this professional should also be responsible for the day to day provision of IDE, whilst senior staff believed that support from an exercise professional was not required beyond initial implementation and that responsibility for the programme might come from another source such as a nurse, non-qualified member of staff or patient.

### Post implementation

Following implementation, responses from both patients and staff regarding exercise were overwhelmingly positive. For both groups observing and experiencing the benefits of IDE was the largest theme that emerged, indicating that although barriers to participation existed these were outweighed by positive experiences.

#### Barriers. Staff workload

Staff workload and lack of time continued to be the most frequently cited barrier to sustaining IDE participation. Four staff members felt that they were unable to adequately supervise exercise sessions, creating concern about patient safety. Seven patients concluded that staff did not prioritize IDE because of their workload, a view confirmed by five staff members.

Large workloads, lack of time and unpredictable shift patterns also led staff to report difficulty attending IDE training sessions or using the knowledge and skills gained from them. This view was confirmed by seven patients who expressed frustration that staff were not as adept at running the programme as exercise professionals because of lack of familiarity.

#### Lack of staff responsibility

Staff and patients believed that there was too much variation in IDE provision due to a lack of responsibility for the programme amongst staff. Seven patients identified the importance of an exercise professional to the initial implementation of the programme but also felt that a dedicated staff member (not necessarily an exercise professional) was important to ongoing success. This individual could act as a ‘coach’, providing feedback, encouragement and support.

#### Facilitators. Positive outcomes of participating in IDE

Post-implementation all patients described experiencing personal improvements as their main motivation to continue. These were primarily enhanced functional abilities (e.g. increased walking capacity), better physiological and psychological health (e.g. blood pressure control, improved mood) and a reduction in symptoms. Such improvements were evident through reassessment, enhanced exercise performance and patients observations of improvements within their daily lives.

Staff were also strongly motivated by these benefits, which they had either observed for themselves or heard about from patients. Positive changes in patients’ moods, improved concordance with dietary and fluid recommendations and reduced symptoms were particularly influential to staff. Observing these benefits dispelled misconceptions about patients’ abilities and boosted staff engagement. All staff positively described how their involvement had made them feel good and how the ethos of the unit had changed to one of health promotion following implementation.

#### Collaboration and teamwork

A collaborative approach between staff members of all grades and disciplines, as well as patients was seen to facilitate the initial implementation and maintenance of the programme. For junior staff, the active involvement and leadership of senior staff was particularly influential. Four staff members also described how patients competent in using the bikes and familiar with the running of the programme had a positive influence.

## Discussion

IDE programmes are rarely adopted in clinic practice despite the high levels of inactivity, morbidity and mortality in the HD population. In order to address this issue effectively, strategies to facilitate IDE implementation and to sustain such programmes are clearly warranted. No existing studies examining barriers and facilitators to IDE have explored the views of both staff and patients using a theory driven approach.

Our study identifies several psychological constructs that may influence IDE implementation and ongoing participation. Techniques aimed at addressing patient and staff beliefs about IDE may be most effective during implementation. Patients indicated their concerns would best be addressed through social influences, whilst staff required increased knowledge and skills. Following implementation, positive outcomes that shaped participants beliefs as well as social influences helped to sustain the programme. Despite this, environment and resource limitations remained the greatest barrier perceived by both groups at all stages

Patient and staff members believed that a better understanding of IDE would facilitate initial participation. Significantly higher exercise participation rates have been reported amongst patients receiving education and support alongside IDE, although no such study exists regarding staff involvement.[42] In practice, increasing patient knowledge and providing opportunities to observe, try and learn about IDE is currently very limited. Within the UK, patient and staff information regarding IDE is typically locally produced, poorly described, unstructured, rarely based upon any theory of learning and behaviour change or subject to any formal evaluation.[8,43–48]Staff have a pivotal influence upon the success of an IDE programme and are well placed to improve patients’ understanding due to frequent contact and strong rapport. However, current programmes rarely utilise trained staff members.[19,20,22,27,28,45] Without formal, high quality education programmes for staff, there is a lack of awareness and confidence about IDE, which may account for the lack of IDE availability and low levels of exercise encouragement that patients receive.[20,27,28]

The success of IDE education programmes may also be influenced by their content and *how* they are delivered, which has not previously been investigated. Peer support from those with personal experience of haemodialysis and IDE participation, as well as the provision of opportunities to experience cycling during treatment may be an effective way of informing patients and enhancing motivation compared to traditional didactic education.[45,49–56] Existing programmes do not offer such opportunities, nor is peer support utilised in a systematic way. Comprehensive peer support has proven to be equivalent to health professional support in other chronic disease populations and may provide more consistent input where staff workload is prohibitive.[22,23,55–57] Workload demands appear to make it difficult for staff to access the training they desired. The provision of interactive e-learning may allow for flexible, convenient staff training, partially addressing this barrier.[58]

Patient and staff attitudes regarding exercise differed markedly in our study. Multiple studies, including ours, have demonstrated that many haemodialysis patients are interested in exercise and believe it beneficial [21,23,26,29]. However, staff perceptions of patients’ interest in and ability to exercise were initially very negative. Other studies highlight that staff focus primarily on patient-related barriers rather than those relating to themselves. [19,20,21,24,27,28] Negative staff perceptions of patients’ ability to exercise may exist to help rationalise a lack of exercise encouragement. Professional assumptions and the resultant behaviours they create have been associated with the failure of new initiatives.[59] Left unaddressed, this may become true for IDE programmes and could become harmful if it leads to withholding of information and opportunities to participate.[21,27,28,60] This study suggests that staff beliefs may successfully be challenged through observation of patients participating in IDE, and this should form an important part of their training.

Previous research identifies that patient participation is primarily sustained by patients observing the benefits of their involvement.[21,23,25] Our study also highlights that the same is true for staff observing the improvements achieved by patients in their care. Such observations were enhanced by the use of assessment. No existing research has highlighted the value of assessment in enhancing long-term patient and staff participation, yet participants within this study viewed it as essential to sustaining participation.[6,22,60] An assessment should include baseline outcomes which are meaningful to patients and staff to increase awareness of the benefits and maintain participation. Currently, haemodialysis staff are untrained in such assessments and they do not form a part of their routine practice. [19,20,27]

Our research also indicates that IDE itself requires greater clarification. The nature of the behaviour being addressed is a domain within the TDF which describes and details the essential characteristics of the task. [30,33] Our study clearly shows that there is confusion regarding the roles of HD staff and exercise professionals in relation to IDE as well as the responsibilities of senior HD staff. This requires urgent clarification as exercise professionals are not routine members of haemodialysis staff. Additionally, clarification of the roles of routine staff should also aid programme implementation and maintenance as senior support was described as a strong catalyst to junior staff engagement at both stages.

Excessive staff workload was an important barrier to the initiation of IDE for both patients and staff. This is strongly supported by other research studies, underlining the importance of addressing this issue as part of defining the nature of IDE. [19,20,21,22,28] Our work shows that staff workload continued to be the primary barrier to sustaining IDE. Renal exercise guidance needs to address staffing and workload issues if IDE programmes are to be widely implemented and successfully sustained.

### Limitations

Our sample is not designed to be statistically representative but rather to purposively explore a range of barriers and facilitators to IDE amongst a diverse range of patients. It was not possible to follow the same patients and staff at the two time points as IDE was not implemented at both units. This is reflected in the decision to use focus groups in phase one in order to capture the ‘collective’ participants’ perceptions rather than to ‘track’ individuals.

## Conclusion

This qualitative study highlights the psychological constructs most influential to implementing and sustaining an IDE programme from the perspectives of both HD patients and staff. Future research should focus upon developing and piloting interventions that utilise techniques for implementation and behaviour change that are linked to these constructs. Our work also clearly highlights the need for the nature of IDE programmes to be clearly defined, including the behaviours and tasks associated with IDE implementation, the roles and responsibilities of different professionals and the prerequisites for the environment in which the programme is to be integrated. Specific IDE guidance that addresses training requirements, staffing levels, roles and skill mix is warranted to fulfill these requirements and may further increase national recognition of the importance of IDE and better facilitate the widespread implementation and long term maintenance of such programmes. [30–31]
